# In Vivo Assessment of Metabolic Abnormality in Alport Syndrome Using Hyperpolarized [1-^13^C] Pyruvate MR Spectroscopic Imaging

**DOI:** 10.3390/metabo11040222

**Published:** 2021-04-06

**Authors:** Nguyen-Trong Nguyen, Eun-Hui Bae, Luu-Ngoc Do, Tien-Anh Nguyen, Ilwoo Park, Sang-Soo Shin

**Affiliations:** 1 Department of Biomedical Science, Chonnam National University, Gwangju 61469, Korea; trongnguyen1408@gmail.com; 2 Department of Internal Medicine, Chonnam National University Medical School and Hospital, Gwangju 61469, Korea; baedak76@gmail.com; 3 Department of Radiology, Chonnam National University Medical School and Hospital, Gwangju 61469, Korea; doluungoc@gmail.com (L.-N.D.); tien.nguyenanh94@gmail.com (T.-A.N.); 4 Department of Artificial Intelligence Convergence, Chonnam National University, Gwangju 61186, Korea

**Keywords:** hyperpolarized ^13^C MRI, Alport syndrome, metabolites, kidney

## Abstract

Alport Syndrome (AS) is a genetic disorder characterized by impaired kidney function. The development of a noninvasive tool for early diagnosis and monitoring of renal function during disease progression is of clinical importance. Hyperpolarized ^13^C MRI is an emerging technique that enables non-invasive, real-time measurement of in vivo metabolism. This study aimed to investigate the feasibility of using this technique for assessing changes in renal metabolism in the mouse model of AS. Mice with AS demonstrated a significant reduction in the level of lactate from 4- to 7-week-old, while the levels of lactate were unchanged in the control mice over time. This reduction in lactate production in the AS group accompanied a significant increase of PEPCK expression levels, indicating that the disease progression in AS triggered the gluconeogenic pathway and might have resulted in a decreased lactate pool size and a subsequent reduction in pyruvate-to-lactate conversion. Additional metabolic imaging parameters, including the level of lactate and pyruvate, were found to be different between the AS and control groups. These preliminary results suggest that hyperpolarized ^13^C MRI might provide a potential noninvasive tool for the characterization of disease progression in AS.

## 1. Introduction

Alport syndrome (AS) is an inherited disease manifested by impaired kidney function, ocular abnormalities, and hearing loss [1]. Mutations of type IV collagen chains, which are major components of the capillary basement membranes, cause Alport syndrome. Normally, embryonic α1α1α2 chain is replaced during development by the mature α3α4α5 chain [2,3]. In Alport patients, this switch is absent, rendering glomerular basement membrane (GBM) more vulnerable to filtration pressure and endoproteolysis [4]. Although there is currently no therapy to prevent end-stage renal disease in these patients, early treatment with renin angiotensin aldosterone system inhibitors was shown to delay end-stage renal disease in patients with Alport syndrome, especially in children with family history [5]. Early and accurate diagnosis is crucial for providing an appropriate treatment option. Although genetic testing proved to be efficient and accurate in detecting genetic abnormality [6], it requires an invasive procedure and a high implementation cost. The development of a noninvasive technique for early characterization of the disease is of clinical importance.

Hyperpolarized carbon-13 (^13^C) magnetic resonance imaging (MRI) is an emerging imaging technique that enables the noninvasive, real-time monitoring of in vivo metabolic processes [7]. This technique was applied to assess kidney function by measuring metabolic processes in various renal conditions, including acute kidney injury [8,9,10], renal tubular injury [11,12], and renal cell carcinomas [13,14,15]. Hyperpolarized ^13^C imaging with pyruvate was applied to humans in prostate, heart, brain, liver, and bone [16,17,18,19,20,21,22,23]. Clinical trials are undergoing using hyperpolarized ^13^C-pyruvate in kidney and it would be possible to translate this study to humans.

The purpose of this study was to explore the potential of hyperpolarized ^13^C MRI for assessing in vivo kidney function in Alport syndrome. We evaluated in vivo metabolism of 4- and 7-week-old mouse model of Alport syndrome and compared the results with wild-type (WT) mice. Overall, our finding identified the changes in renal metabolism, which were associated with the disease progression of Alport syndrome. The results from this study might provide a noninvasive method for monitoring renal function that is associated with disease progression in Alport syndrome.

## 2. Results

**Figure 1:**
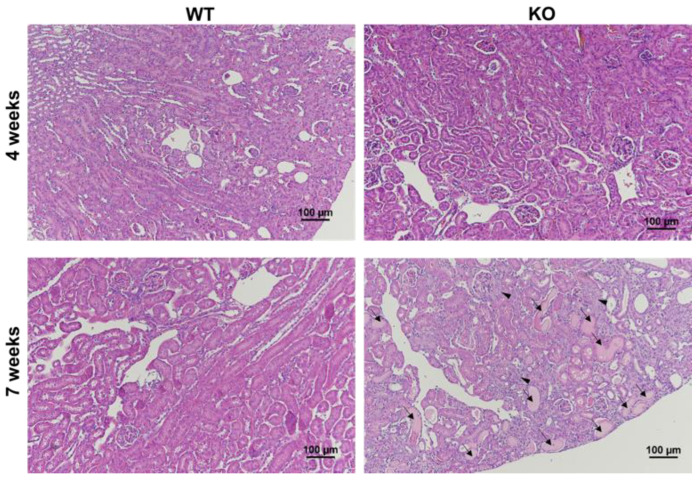
Renal histological analysis showed kidney diseases in the mouse with Alport syndrome. Hematoxylin and eosin (H&E) staining in the kidney section revealed glomerulosclerosis (arrowhead) and tubular dilatation (arrow) in the 7-week-old KO mouse, compared to the other group. The normal glomerular (arrow head) and tubular (arrow) regions of the 4-week-old KO mouse are also shown for comparison. Magnification ×100.

The knock-out (KO) Col4α3^−/−^ animals that were used to create the mouse model of Alport syndrome demonstrated a progressive kidney disease. Table 1 shows the summary of body and kidney weights, and the comparison of albumin-to-creatinine ratio (ACR), which is a measure of kidney disease and failure. The ACR of KO mice significantly increased from 198.3 milligrams of albumin per gram of creatinine (mg/gCr) at 4-week-old to 11,423.3 mg/gCr at 7-week-old (*p* = 0.029), while the ACRs of WT mice were similar over the same period (8.4 mg/gCr at 4-week-old and 13.3 mg/gCr at 7-week-old, *p* = 0.11). In addition, the ACRs of KO mice were significantly higher than those of WT mice at both time-points (Table 1). These findings suggest that the KO mice presented abnormal kidney function at an early stage of the disease at 4-week-old, which was exacerbated over time. Similarly, the pathological analysis of the kidney tissues revealed the progression of disease in the KO mice over time with extensive glomerulosclerosis and tubular dilatation in the H&E slide of the 7-week-old KO mouse (Figure 1).

**Figure 2:**
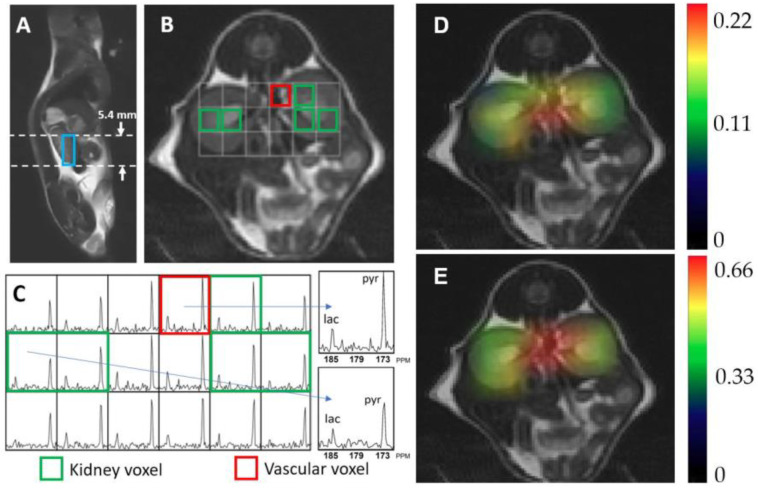
Representative hyperpolarized ^13^C magnetic resonance spectroscopic imaging data, showing a sagittal (**A**) and an axial T2-weighted image (**B**) of kidney, and the corresponding ^13^C magnetic resonance spectra (**C**). Each spectroscopic voxel exhibits signals from the injected substrate, pyruvate (pyr, 172 ppm), and its metabolic product, lactate (lac, 184 ppm). Colormap overlay images are shown for the normalized lactate (**D**) and pyruvate (**E**).

Figure 2 shows exemplary hyperpolarized ^13^C metabolic imaging data. Figure 2A,B are anatomical images of a 7-week-old WT mouse in a sagittal and an axial plane, respectively. The blue box in Figure 2A represents the 5.4 mm slice thickness of the ^13^C magnetic resonance spectroscopic imaging (MRSI) data that were used to quantify the ^13^C metabolic signals. The corresponding axial image in Figure 2B exhibits the array of ^13^C MRSI voxels with an in-plane resolution of 2 × 2 mm, which contain metabolic information in each voxel location. The voxels used to quantify metabolic signals in kidneys and blood vessels are identified with green and red outliners, respectively. Figure 2C shows the corresponding ^13^C MRSI data demonstrating hyperpolarized ^13^C pyruvate signal and its metabolic product, lactate. Alanine and bicarbonate were not detected in kidney, presumably due to the relatively small level of these signals compared to other metabolites and the small size of voxel (2 × 2 × 5.4 mm). 

**Figure 3:**
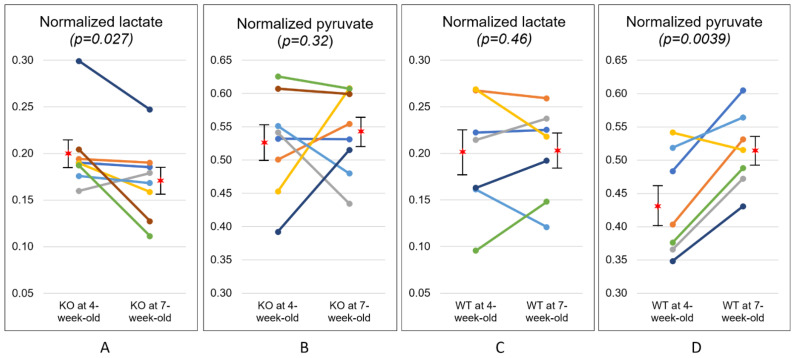
Temporal changes in metabolism at two time-points. The level of normalized lactate significantly decreased with disease progression in the knock-out (KO) mice (**A**), while the level of normalized pyruvate significantly increased over time in the wild-type (WT) mice (**D**). The level of normalized pyruvate of the KO mice (**B**) and normalized lactate of the WT mice (**C**) were comparable at two time points. The star symbols represent the mean values and the error bars indicate the standard error of the mean.

Table 2 summarizes the results from longitudinal data where the temporal changes in metabolism from 4- to 7-week-old were analyzed for each animal. Normalized lactate, normalized pyruvate, and the ratio of lactate to pyruvate (Lac/Pyr) at 4-week-old were compared with those at 7-week-old for each group. The KO mice at 4-week-old exhibited a normalized lactate of 0.20 ± 0.04, which was significantly higher than the corresponding value at 7-week-old (0.17 ± 0.04, *p* = 0.027) (Figure 3A). Similarly, Lac/Pyr in the KO group showed a trend toward decrease over time (0.40 ± 0.15 at 4-week-old; 0.33 ± 0.10 at 7-week-old, *p* = 0.075). In contrast, the levels of normalized lactate in the WT mice were comparable between 4-week-olds and 7-week-olds (0.19 ± 0.06 and 0.20 ± 0.05, respectively, *p* = 0.46). The level of normalized pyruvate in the WT mice significantly increased from 0.43 ± 0.08 at 4-week-old to 0.52 ± 0.06 at 7-week-old (*p* = 0.0039, Figure 3D), while the KO mice exhibited similar levels of normalized pyruvate between two time-points (0.53 ± 0.08 at 4-week-old; 0.54 ± 0.06 at 7-week-old, *p* = 0.32). Lac/Pyr in the WT group demonstrated a significant decrease over time (0.47 ± 0.15 at 4-week-old; 0.40 ± 0.11 at 7-week-old, *p* = 0.016), which was mostly induced by the elevated level of pyruvate over the same period of time.

**Figure 4:**
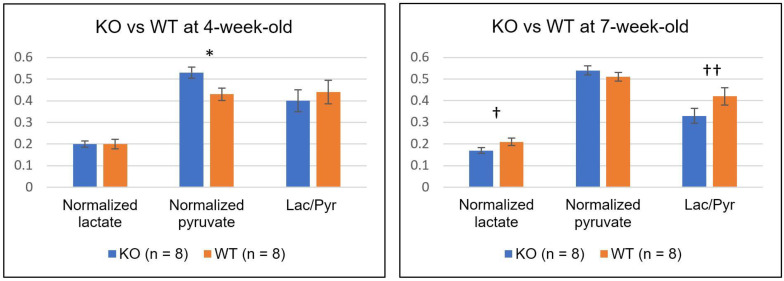
The comparison of normalized lactate, normalized pyruvate, and the ratio of lactate to pyruvate (Lac/Pyr) between the knock-out (KO) and wild-type (WT) mice at 4- (**left**) and 7-week-old (**right**). The level of normalized pyruvate in the KO mice was significantly higher than that in the WT mice at 4-week-old (* *p* = 0.040). At 7-week-old, the KO mice showed relatively lower levels of both normalized lactate (^†^ *p* = 0.064) and Lac/Pyr (^††^ *p* = 0.056) compared to the WT mice. Error bars indicate the standard error of the mean.

Table 3 and Figure 4 summarize the comparison between the two groups of mice at each time-point. At 4-week-old, the level of normalized pyruvate in the KO group (0.53 ± 0.08) was significantly higher than that in the WT group (0.45 ± 0.08, *p* = 0.040). However, both levels of normalized lactate and Lac/Pyr were similar between them (0.20 ± 0.04 and 0.19 ± 0.07 for the normalized lactate of KO and WT, respectively, *p* = 0.34; 0.40 ± 0.15 and 0.44 ± 0.17 for the Lac/Pyr of KO and WT, respectively, *p* = 0.31). At 7-week-old, the KO group demonstrated a strong trend toward a lower level of both normalized lactate (0.17 ± 0.04) and Lac/Pyr (0.33 ± 0.10), as compared to the WT group (0.21 ± 0.05 for normalized lactate, *p* = 0.064; 0.42 ± 0.12 for Lac/Pyr, *p* = 0.056). In contrast, the levels of normalized pyruvate were comparable between the two groups at 7-week-old.

**Figure 5:**
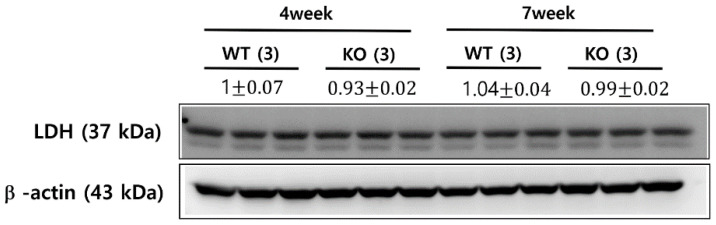
LDH expression levels from Western blot analysis. The knock-out (KO) and wild-type (WT) mice at 4- and 7-week-old exhibited comparable levels of LDH expression between all groups of mice. The values are reported as mean ± standard deviation.

Lactate dehydrogenase (LDH) is a cytosolic enzyme that catalyzes the conversion of pyruvate to lactate. Previous hyperpolarized ^13^C imaging studies of drug-treated tumors showed the association between the reduced pyruvate-to-lactate conversion and a number of factors, including a decreased LDH expression and a loss of LDH activity [24,25]. LDH expression levels were assessed using a Western blot analysis from age- and sex-matched additional animals of KO and WT mice at 4-week-old and 7-week-old. Although the 7-week-old KO mice demonstrated a significantly decreased level of lactate compared to the 4-week-old KO mice (Figure 3A), the two groups exhibited comparable levels of LDH expression (Figure 5). Similarly, although the 7-week-old KO mice showed a smaller level of normalized lactate, compared to the 7-week-old WT mice (Figure 4), the levels of LDH expression were similar among these two groups of animals (Figure 5).

**Figure 6:**
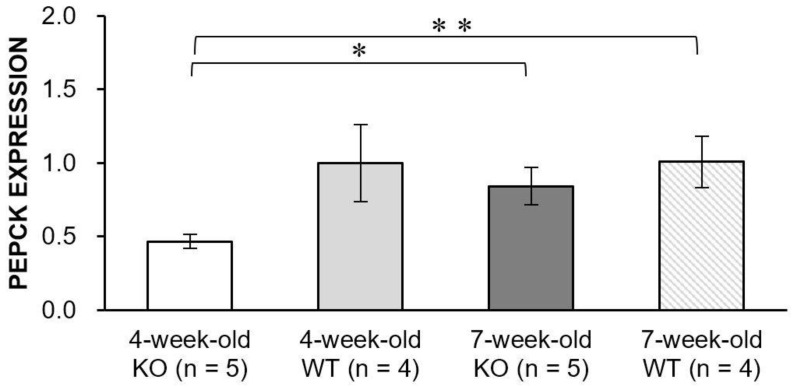
Bar graphs showing PEPCK expression levels in the knock-out (KO) and wild-type (WT) mice at 4- and 7-week-old. The level of PEPCK expression significantly increased with disease progression in the KO mice (* *p* = 0.024). In addition, the level of PEPCK expression in the 4-week-old KO mice was significantly smaller than that of the 7-week-old WT mice (** *p* = 0.010). Error bars indicate standard error of the mean.

We measured the expression level of phosphoenolpyruvate carboxykinase (PEPCK), which is considered to be the rate-limiting enzyme of gluconeogenesis (Figure 6). The PEPCK expression level of the WT mice at 4-week-old was similar to that at 7-week-old (*p* = 0.98). In contrast, the KO mice exhibited a significantly increased level of PEPCK expression at 7-week-old compared to 4-week-old (*p* = 0.024), which might indicate that the KO mice might have possessed an impaired PEPCK action and the level of gluconeogenesis might have changed with the disease progression in Alport syndrome.

## 3. Discussion

Based on its ability to evaluate pathological condition by measuring real-time in vivo metabolism, hyperpolarized ^13^C MRI was studied for various pathological conditions such as cancer, cardiovascular, and liver diseases [7,26,27,28,29]. Although several previous studies investigated the feasibility of using this technique for monitoring kidney function, most of these studies focused on studying diabetes-induced renal failure [30,31,32] or acute kidney injury, such as renal ischemia [8,9,11]. In this study, we applied hyperpolarized ^13^C-pyruvate MRI to monitor renal function in the mouse model of Alport syndrome, a rare genetic disorder characterized by progressive kidney disease. ^13^C-labeled pyruvate was injected, and hyperpolarized metabolic MRI data were acquired at two time-points in the progression of the disease in both the KO and WT models. We aimed to evaluate early temporal change in kidney function by assessing hyperpolarized pyruvate-to-lactate metabolism in Alport syndrome and comparing it to the control group.

The mice with Alport syndrome demonstrated a reduction in lactate production with the progression of disease, while the levels of lactate were unchanged in the WT mice over time. Another finding from the longitudinal assessment was a significant increase in the level of pyruvate over time in the kidney of the WT mice, which might be due to an increased level of kidney perfusion with the development of healthy kidney. This was consistent with a significant increase in the body weight of the WT mice over time (15.5 ± 1.4 g at 4-week-old imaging and 20.0 ± 2.1 g at 7-week-old imaging, *p* < 0.01). Similarly, the kidney volume of the WT mice measured from the ^1^H anatomical images demonstrated a significant increase over time (115.8 ± 4.3 mm^3^ at 4-week-old imaging and 148.6 ± 11.8 mm^3^ at 7-week-old imaging, *p* < 0.05). The increased body and kidney size might have affected the level of perfusion in kidney, which could allow an increased level of pyruvate in older mice. However, the levels of pyruvate were similar for the KO mice between 4 and 7 week, even though the body weight and kidney volume of KO mice significantly increased over time (body weight, 17.2 ± 2.3 g at 4-week-old imaging and 22.0 ± 3.4 g at 7-week-old imaging, *p* < 0.01; kidney volume, 129.5 ± 5.7 mm^3^ at 4-week-old imaging and 181.8 ± 13.4 mm^3^ at 7-week-old imaging, *p* < 0.01). Interestingly, while both the body weight and kidney volume were comparable between the KO and WT mice at 4 week, the 4-week-old KO mice exhibited a significantly elevated level of pyruvate compared to the 4-week-old WT mice and this elevated level of pyruvate in the KO mice persisted over time.

Our imaging findings suggest that the kidney damage elicited by disease progression of Alport syndrome might have led to a reduction in substrate utilization and the subsequent reduction in hyperpolarized ^13^C pyruvate-to-lactate conversion. Several possible explanations exist for the observed metabolic patterns. One possible explanation might be the elevated usage of lactate as a renal gluconeogenic precursor. PEPCK is a detectable enzyme that is used in the metabolic pathway of gluconeogenesis and is widely used as a marker for gluconeogenesis [33]. Our PEPCK expression analysis showed a significantly increased renal PEPCK expression level in the KO mice at 7-week-old, compared to that at 4-week-old, which supports a significant elevation of renal gluconeogenesis in the KO mice with the progression of disease. Gluconeogenesis is a metabolic process that leads to the generation of glucose from certain carbon substrates. Although gluconeogenesis primarily occurs in the liver, the kidney provides a substantial contribution of gluconeogenesis under both physiological and pathological conditions, and preferentially uses lactate as one of the main gluconeogenic precursors [34,35]. Since lactate is quantitatively the biggest source of substrate for gluconeogenesis, especially in the kidney [35], it is likely that a small amount of lactate remains in the kidney under the high level of gluconeogenesis. It was shown that the observed hyperpolarized ^13^C pyruvate and lactate signals mostly reflect a ^13^C label exchange between the endogenous metabolite pools [36]. The reduced level of hyperpolarized ^13^C lactate in the 7-week-old KO mice in comparison to the 4-week-old KO mice, therefore, might represent the decreased size of the endogenous lactate pool caused by the high utilization of lactate as a gluconeogenic precursor in the kidney. It is difficult to elucidate the exact mechanisms that resulted in the observed decrease of lactate levels, such as downstream enzymatic changes that accompany renal gluconeogenesis. However, our results suggest that the progression of disease in the mice with Alport syndrome might trigger the gluconeogenic pathway that converts lactate to glucose and might result in a decreased lactate pool size and a consequent reduction in pyruvate-to-lactate exchange in the 7-week-old KO mice.

Another observation that is worth noting is the comparable levels of LDH expression between the KO and WT mice at both time-points. LDH catalyzes the reversible process of pyruvate to lactate. Since an elevated aerobic glycolysis and the consequent increase in lactate production, famously known as the Warburg effect, is characteristics of many cancers, a number of studies of hyperpolarized cancer imaging showed the association between the changes in the level of LDH and the resulting changes in hyperpolarized pyruvate-to-lactate conversion [7]. We measured the levels of LDH expression to test if there were any associations between the observed reduced level of lactate in the 7-week-old KO mice, as compared to the 4-week-old KO mice. However, they were not correlated. A few previous studies showed a similar observation where the changes in lactate production were not linked to the changes in the levels of LDH, but with other metabolic enzyme, such as pyruvate kinase PKM2 [37] or pyruvate dehydrogenase [38,39], in the context of other diseases, including brain tumor, neuroinflammation, and traumatic brain injury. A further investigation is needed to explore the association between our imaging findings and the possible mechanistic explanation. Although very little is known about genetic variations that modulate metabolites in Alport syndrome or downstream energetics, a recent study reported that renal tubules in Alport mice showed dysmorphic mitochondria with defective bioenergetics, which suggests that Alport pathology is associated with impaired mitochondrial respiration [40]. A follow-up study is currently being planned, which will investigate the use of other ^13^C molecules or isotopomers [41,42,43,44,45] for probing mitochondrial metabolism and assessing the associated biochemical mechanism that might accompany Alport syndrome.

The results from the urinary microalbuminuria analysis indicated that the KO mice used in our study exhibited early kidney disease, which progressed over time. The significantly elevated level of pyruvate in the 4-week-old KO mice compared to the WT mice with the same age implied a relative reduction in substrate utilization in the KO mice, as compared to the WT mice at 4-week-old. Abnormal renal function in the KO mice might have prevented the kidney from undergoing normal metabolism and could have resulted in a significantly decreased utilization of substrate. A recent study observed a similar reduction in pyruvate utilization that was associated with kidney damage caused by ischemic reperfusion injury [11]. In addition, the 7-week-old KO mice produced the relatively smaller level of lactate compared to the 7-week-old WT mice, which might be associated with abnormal metabolic function caused by extensive kidney damages that occurred with the progression of disease. The histological analysis of the kidney from the 7-week-old KO mouse revealed a large area of glomerulosclerosis and tubular dilatation, which are typical signs of renal dysfunction. Although further experiments are necessary to investigate the underlying mechanism, these findings suggest that hyperpolarized ^13^C pyruvate MRI has a potential to be used as a noninvasive tool for early detection and monitoring of the abnormal renal function in Alport syndrome.

Previous hyperpolarized ^13^C MRI studies investigated the metabolic response in various pathological conditions of kidney [8,9,11,13]. A number of studies demonstrated the feasibility of using this technique in assessing oxidative stress and mitochondrial activity associated with renal ischemia reperfusion injury [8,9,11]. Other studies applied this technique to assess conditions associated with hepatic or renal gluconeogenesis, using either isolated perfused organs [46,47] or in vivo models [47,48,49,50,51]. A majority of these studies focused on pre-clinical models of fasting or diabetes, as they represent most well-known conditions where gluconeogenesis occurs. To our knowledge, our study was the first effort to apply the hyperpolarized ^13^C metabolic imaging method to a genetic disease model in the kidney. The Alport syndrome model used in this study developed an elevated level of gluconeogenesis with disease progression, which was consistent with the significant reduction in the level of lactate over time in the KO mice.

Several limitations exist in our study. Although our preliminary results appear to be promising in probing temporal changes in metabolism with disease progression in Alport syndrome and assessing difference in metabolic patterns between the KO and WT mice, they should be interpreted with the potential limitations of the methodology used to acquire the data. Although the ^13^C MRSI sequence was able to obtain a relatively small spatial resolution (2 × 2 mm in-plane resolution with 5.4 mm slice thickness), the size of the spectroscopic voxel was relatively big, compared to the size of mouse kidney. The observed metabolic signals likely represent collective metabolisms from cortex, medulla, vasculature, and adjacent tissues. Renal medulla and cortex were shown to present disparate metabolic patterns under certain conditions [52,53]. The acquisition of ^13^C hyperpolarized MRI data with a higher spatial resolution [54] might be considered to characterize distinct metabolism in different renal compartments. Another limitation of this study is the lack of mechanistic backgrounds for the observed metabolic imaging findings. Alport syndrome is a rare genetic disease and most of the previous studies focused on elucidating the underlying mechanisms associated with its genetic phenotype or exploring a new therapeutic target [55,56,57]. Very little is known about the specific cellular metabolisms that accompany the Alport syndrome. Although we tried to assess the mechanistic basis for the observed imaging findings by evaluating the PEPCK and LDH expression levels, further studies are necessary for understanding the underlying cellular mechanisms that take place in the progression of Alport syndrome and relating them to the experimental results from the hyperpolarized ^13^C imaging studies.

## 4. Materials and Methods

### 4.1. Subjects

All study procedures were approved by the Institutional Animal Care and Use Committee. Eight male KO Col4*α*3^−/−^ (body weight = 17.2 ± 2.3 g at 4-week-old imaging and 22.0 ± 3.4 g at 7-week-old imaging) and nine male WT mice on a congenic 129X1/SvJ background (body weight = 15.5 ± 1.4 g at 4 week and 20.0 ± 2.1 g at 7 week) from the Jackson Laboratory (Bar Harbor, ME, USA) were included to obtain the metabolic imaging data. The hyperpolarized ^13^C MR exams were performed when the mice were 4- and 7-weeks old. One WT mouse was scanned at 4-week-old only and the other WT mouse at 7-week-old only. Additional KO (n = 16) and WT (n = 14) mice were included to assess the levels of LDH and PEPCK expression, and the urinary microalbuminuria level. The mice were raised with typical food and free access to water at the animal facility in Chonnam National University, Gwangju, Korea.

### 4.2. ^1^H and ^13^C Imaging Examination

The ^13^C and ^1^H imaging data were acquired on a 3 Tesla clinical MRI scanner (Discovery MR750; GE Healthcare, Milwaukee, WI, USA), integrated with a package of multinuclear spectroscopy and a ^1^H/^13^C dual mouse coil (RAPID biomedical, Rimpar, Germany). The animals were fasted for 8 h prior to the imaging studies, and all imaging exams were performed at approximately the same time of the day, in order to minimize any physiological variations. Throughout the imaging exam, mice were properly positioned under the heating pad inside the coil, while receiving continuous delivery of anesthesia (approximately 1.5%). A mixture of 18 µL of [1-^13^C]-pyruvate, 15 mmol/L OX063 trityl radical (GE Healthcare), and 1.5 mmol/L Gd-DOTA was polarized in a HyperSense^®^ DNP polarizer (Oxford Instruments, Abingdon, UK) at 3.35 T and 1.4 K by irradiation with 94.1 GHz microwave, following the manufacture’s instruction. After approximately 1 h of polarization, the mixture was immediately dissolved in a buffer with 5.96 g/L Tris (40 mmol/L), 4.00 g/L NaOH (100 mmol/L), 0.1 g/L ethylenediaminetetraacetic acid, and 2.9 g/L Sodium Chloride (NaCl). The final solution with a volume of 0.3 mL and a pH of approximately 7.5 was rapidly injected into the tail vein of the mice over a 15 s duration.

The following imaging data were acquired in every MRI experiment—high resolution axial T2-weighted image (TE, 100 ms; TR, 4000 ms; FOV, 6 mm; matrix size, 192 × 192; 1-mm section thickness; and NEX, 7), compressed-sensing ^13^C 3D MRSI data (echo time/repetition time = 140/215 ms, 20 × 16 × 16 matrix, 2 ×2 × 5.4 mm spatial resolution, 30^0^ flip angle) with a centric k-space encoding, and flyback echo-planar readout on the z-axis [58], at 24 s from the start of the injection.

### 4.3. ^13^C Data Processing and Analysis

The methods for processing ^13^C data were described previously [58]. In brief, the raw data were rearranged to a 4D array, to which a 4D Fourier transform was applied to reconstruct it to a 3D spatial-spectral array of ^13^C spectra. A linear phase correction was performed in the z-direction to correct for the offset in k-space. These steps were performed using in-house written scripts and built-in functions in Matlab R2017a (Mathworks, Natick, MA, USA). For the quantification of ^13^C metabolites, lactate, pyruvate, alanine, and total carbon (a sum of peak heights from lactate, pyruvate, alanine, and pyruvate-hydrate) signals were obtained from each voxel, using the magnitude spectra. This step was performed by the semi-automated quantification process in the Spectroscopic Image Visualization and Computing (SIVIC) package [59]. The lactate and pyruvate signals were then exported as metabolite maps, using the built-in function in SIVIC, imported to Matlab as a 3D array, and normalized by the maximum total carbon signal in blood vessels. The voxel for the blood vessels, which contained the highest pyruvate signal, represented the renal arteries and veins, and were typically located in the middle of two kidneys. The normalized lactate, pyruvate, and the ratio of lactate to pyruvate in the kidney voxels, which were defined as voxels containing larger than 90% kidney in volume, were compared between the KO and WT groups at each time-points using an unpaired *t*-test. The temporal changes in metabolism were evaluated for each group using a paired *t*-test. The information for group and time-points were blinded for these analysis. In order to measure the change in kidney size, the kidney volume was measured from ^1^H axial T2-weighted image using ImageJ [60]. The first author (N.T.N), who had approximately 2-year experience with hyperpolarized ^13^C imaging, acquired the data and performed the data analysis with help from other authors (L-N.D. and T.A.N) and under the supervision of the corresponding authors (I.P. and S.S.S.) who had extended experience with hyperpolarized imaging studies.

### 4.4. Urinary Microalbuminuria Measurement

Urine of mice was collected by maintaining mice in individual metabolic cages for 3 days. Urine samples were centrifuged at 8000× *g* for 5 min, immediately after collection. Urinary microalbumin was measured using the turbidimetric immunoassay method (Olympus AU 5431, Toshiba TBA-200FR autoanalyzer, Tokyo, Japan), while urinary creatinine was measured using the Jaffe method. Urinary albumin excretion was estimated as the albumin-to-creatinine ratio in milligrams of albumin per gram of creatinine.

### 4.5. Histology Evaluation

After euthanizing the animals, mouse kidneys were excised, fixed in phosphate-buffered 10% formalin, embedded in paraffin, and stained with hematoxylin and eosin (H&E) for histological examination.

### 4.6. Western Blot Analysis of LDH

To measure the LDH expression level, kidneys were resected from 4- (n = 3) and 7-week-old (n = 3) KO mice, as well as 4- (n = 3) and 7-week-old (n = 3) WT mice. Mouse kidney tissues were washed with ice-cold PBS two times. A total of 50 μL per mg of renal tissue was placed in modified RIPA buffer (150 mM sodium chloride, 50 mM Tris-HCl (pH 7.4), 1 mM EDTA, 1% *v*/*v* Triton-X 100, 1% *w*/*v* sodium deoxycholic acid, and 0.1% *v*/*v* SDS). Tissues were then sonicated two times for 10 s, and incubated on ice for 30 min. Following centrifugation at 18,000× *g* for 10 min, supernatant was transferred to a tube with a loading buffer and boiled for 5 min. Proteins in tissue lysates were separated by 6–12% SDS-PAGE gel, blotted onto the PVDF membrane and detected with an enhanced chemiluminescence system (ECL) kit (Millipore Corp., Billerica, MA, USA). Densitometry was calculated by the Scion Image software (Scion Corp, Frederick, MD, USA). The following primary antibodies were used for Western blot analyses—LDH (Cell Signaling, Danvers, MA, USA) and β–actin (Sigma-Aldrich, St. Louis, MO, USA).

### 4.7. Real-Time PCR for Evaluation of PEPCK Expression Levels

To test the rate of gluconeogenesis, PEPCK expression levels were measured from 4- (n = 5) and 7-week-old (n = 5) KO mice, as well as 4- (n = 4) and 7-week-old (n = 4) WT mice RNA was extracted from snap-frozen kidney tissue, using the RNeasy Mini kit (Qiagen Canada, Mississauga, ON, Canada). Isolated RNA was reversely transcribed into cDNA with the QuantiTech Reverse Transcription kit (Qiagen), which was subsequently used in real-time PCR (run on ABI Prism 7900; Applied Biosystems, Foster City, CA, USA). Mouse primer sets (Bioneer Corporation, Daejeon, Korea) were purchased for the following genes—PEPCK (forward: 5′-AAG GAA AAC GCC TTG AAC CT-3′, reverse 5′-GTA AGG GAG GTC GGT GTT GA-3′).

## 5. Conclusions

In summary, this study demonstrated the potential of using hyperpolarized ^13^C MRI for assessing temporal changes in renal metabolism in Alport Syndrome. The decrease in the level of lactate with disease progression was consistent with the increased PEPCK expression levels. In addition, the difference in metabolisms observed between the KO and WT mice might be used as a noninvasive tool for monitoring and assessing abnormal renal metabolism with the progression of Alport syndrome. Our preliminary findings suggest that this technique might provide important information regarding the characterization of Alport syndrome and warrant further studies to validate our findings and provide mechanistic evidence.

## Figures and Tables

**Table 1:** Comparison of body weight (BW), kidney weight (KW), and albumin-to-creatinine ratio (ACR) between the wild type (WT) and knock-out (KO) mice at 4- and 7-week-old. The KO mice exhibited a significantly increased level of ACR over time, which is a marker for abnormal kidney function, and possessed significantly higher levels of ACR compared to the WT mice at both 4 and 7 weeks. Both the KO and WT mice demonstrated significant increases of their BW, KW and the ratio of KW to BW (KW/BW) over time. The KO mice showed a significantly higher level of KW/BW compared to the WT mice at 7 weeks. Values are reported as means ± standard error of the mean. BW and KW were recorded at the time of euthanasia for PEPCK analysis. * *p* < 0.05 between 4-week-old KO vs. 7-week-old KO; ** *p* < 0.05 between 4-week-old WT vs. 7-week-old WT, ^†^ *p* < 0.05 between 4-week-old KO vs. 4-week-old WT; ^‡^ *p* < 0.05 between 7-week-old KO vs. 7-week-old WT. All statistical tests were performed using an unpaired *t*-test.

	4 Weeks		7 Weeks	
	KO (n = 5)	WT (n = 4)	KO (n = 5)	WT (n = 4)
Body weight (g)	16.2 ± 0.3 *	16.6 ± 0.4 **	19.4 ± 0.6 *	21.5 ± 0.6 **
Kidney weight (g)	0.13 ± 0.006 *	0.12 ± 0.006 **	0.17 ± 0.009 *	0.16 ± 0.005 **
KW (g)/BW (kg)	7.69 ± 0.21 *	6.92 ± 0.20 **	8.96 ± 0.18 *^‡^	7.62 ± 0.11 **^‡^
ACR (mg/g Creatine)	198.3 ± 61.4 *^†^	8.4 ± 1.4 ^†^	11,423.3 ± 5962.4 *^‡^	13.3 ± 2.0 ^‡^

**Table 2:** Temporal changes in metabolism at two time-points. The level of normalized lactate, normalized pyruvate, and the ratio of lactate to pyruvate (Lac/Pyr) are compared between the knock-out (KO) and wild-type (WT) mice at 4- and 7-week-old. Normalized lactate in the KO mice showed a significant reduction over time (* *p* < 0.05), while Lac/Pyr showed a strong trend toward reduction over time in the KO mice (^†^ *p* = 0.075). Normalized pyruvate in the KT mice significantly increased over time (** *p* = 0.0039), while Lac/Pyr showed a strong trend toward reduction over time in the WT mice (^†^ *p* = 0.075). All statistical tests were performed using a paired *t*-test. The values are reported as mean ± standard deviation.

		Normalized Lactate	Normalized Pyruvate	Lac/Pyr
KO (n = 8)	4-week	0.20 ± 0.04 *	0.53 ± 0.08	0.40 ± 0.15 ^†^
	7-week	0.17 ± 0.04 *	0.54 ± 0.06	0.33 ± 0.10 ^†^
WT (n = 7)	4-week	0.19 ± 0.06	0.43 ± 0.08 **	0.47 ± 0.15 *
	7-week	0.20 ± 0.05	0.52 ± 0.06 **	0.40 ± 0.11 *

**Table 3:** Comparison of metabolism between the knock-out (KO) and wild-type (WT) mice. The levels of normalized lactate, normalized pyruvate, and the ratio of lactate to pyruvate (Lac/Pyr) were compared between the two groups of mice at 4- and 7-week-old. Normalized pyruvate in the KO mice was significantly higher than that in the WT mice at 4-week-old (* *p* < 0.05). At 7 week, the KO mice showed a strong trend toward a lower level of normalized lactate (^†^ *p* = 0.064) and Lac/Pyr (^††^ *p* = 0.056) compared to the WT mice. All statistical tests were performed using an unpaired *t*-test. The values are reported as mean ± standard deviation.

		Normalized Lactate	Normalized Pyruvate	Lac/Pyr
4-week	KO (n = 8)	0.20 ± 0.04	0.53 ± 0.08 *	0.40 ± 0.15
	WT(n = 8)	0.19 ± 0.07	0.45 ± 0.08 *	0.44 ± 0.17
7-week	KO (n = 8)	0.17 ± 0.04 ^†^	0.54 ± 0.06	0.33 ± 0.10 ^††^
	WT (n = 8)	0.21 ± 0.05 ^†^	0.51 ± 0.06	0.42 ± 0.12 ^††^

## Data Availability

The data presented in this study may be available upon reasonable request from the corresponding authors.
